# Unexpected pancreatic mixed neuroendocrine-nonneuroendocrine neoplasms (MiNEN)—reflection on a case report

**DOI:** 10.1093/jscr/rjae026

**Published:** 2024-02-03

**Authors:** Catarina Alexandra Quintas Baía, Alexandre Sousa, Fernanda Sousa, Pedro Santos, Ana Isabel Varelas, Luís Pedro Afonso, Joana Monteiro, José Manuel Fernandes, Lúcio Lara Santos, Joaquim Abreu de Sousa

**Affiliations:** Surgical Oncology Department, Portuguese Oncology Institute of Porto (IPO Porto), Porto Comprehensive Cancer Centre (P.CCC), 4200-072 Porto, Portugal; Surgical Oncology Department, Portuguese Oncology Institute of Porto (IPO Porto), Porto Comprehensive Cancer Centre (P.CCC), 4200-072 Porto, Portugal; Surgical Oncology Department, Portuguese Oncology Institute of Porto (IPO Porto), Porto Comprehensive Cancer Centre (P.CCC), 4200-072 Porto, Portugal; General Surgery Department, Torres Vedras Hospital, West Hospital Centre, 2560-295 Torres Vedras, Portugal; Pathology Department, Portuguese Oncology Institute of Porto (IPO Porto), Porto Comprehensive Cancer Centre (P.CCC), 4200-072 Porto, Portugal; Pathology Department, Portuguese Oncology Institute of Porto (IPO Porto), Porto Comprehensive Cancer Centre (P.CCC), 4200-072 Porto, Portugal; Medical Oncology Department, Portuguese Oncology Institute of Porto (IPO Porto), Porto Comprehensive Cancer Centre (P.CCC), 4200-072 Porto, Portugal; Surgical Oncology Department, Portuguese Oncology Institute of Porto (IPO Porto), Porto Comprehensive Cancer Centre (P.CCC), 4200-072 Porto, Portugal; Surgical Oncology Department, Portuguese Oncology Institute of Porto (IPO Porto), Porto Comprehensive Cancer Centre (P.CCC), 4200-072 Porto, Portugal; Surgical Oncology Department, Portuguese Oncology Institute of Porto (IPO Porto), Porto Comprehensive Cancer Centre (P.CCC), 4200-072 Porto, Portugal

**Keywords:** mixed neuroendocrine-nonneuroendocrine neoplasm, pancreas, neuroendocrine, mixed neoplasm

## Abstract

The authors present a case involving a 51-year-old male who was diagnosed with a 4-cm mass in the body of the pancreas, initially suspected to be a ductal adenocarcinoma due to an elevated Ca 19.9 during routine analysis. Subsequent imaging studies confirmed a resectable disease without suspicious lymph nodes or distant metastasis, leading to the proposal of surgery. The patient underwent a laparoscopic distal splenopancreatectomy, which was uneventful. The histopathological examination revealed a 3.7-cm pancreatic mixed neuroendocrine neoplasia (MiNEN) with a predominant high-grade ductal adenocarcinoma component and a concurrent high-grade neuroendocrine carcinoma, with negative margins. Two lymph node metastases were identified, each representing metastasis of one of the components. The tumor was classified as pT2N1M0. Currently, the patient is undergoing chemotherapy with FOLFIRINOX. This case prompts reflection on the optimal treatment strategy for pancreatic MiNEN and raises the question of how the preoperative diagnosis could influence the patient’s outcome.

## Introduction

Mixed neuroendocrine-nonneuroendocrine neoplasms (MiNEN) are rare tumors characterized by the presence of two morphologically distinct neoplastic components: a neuroendocrine component and a non-neuroendocrine component, each constituting at least 30% of the entire tumor [1]. The concept of MiNEN expanded on the definition of mixed-adenocarcinoma neuroendocrine carcinoma (MANEC) because the latter failed to encompass the full spectrum of this neoplasm, with combinations of different histological components and grades [1].

Pancreatic MiNEN is a rare tumor, constituting < 0.5% of all pancreatic resections [2]. It encompasses various histological combinations resulting in high-grade neoplasms through the combination of neuroendocrine carcinoma (NEC) with ductal adenocarcinoma or acinar carcinoma, as well as intermediate-grade neoplasms involving G1/G2 neuroendocrine tumor [3]. The most prevalent type of pancreatic MiNEN is mixed ductal adenocarcinoma-NEC (adeno-MiNEN). It primarily affects males with a mean age of 60 years. Commonly located in the pancreatic head, 54% are diagnosed at stage III [2, 4]. In the majority of cases, diagnosis is typically established only after resection surgery, owing to the inherent heterogeneity of the tumor which makes it difficult for biopsy material to fully capture both components [2, 4, 5]. While there is no complete consensus on the treatment of MiNEN, curative-intent surgery is recommended as the first treatment of choice, followed by adjuvant treatment for high and intermediate-grade MiNEN [4–7]. Given its heterogeneity, prognosis depends largely on tumor staging and the most aggressive component [2, 3, 7]. Despite significant variation in mean survival across different studies (ranging from 12 to 88 months), MiNEN is associated with a better prognosis than ductal adenocarcinoma [2, 4, 7].

## Case report

**Figure 1:**
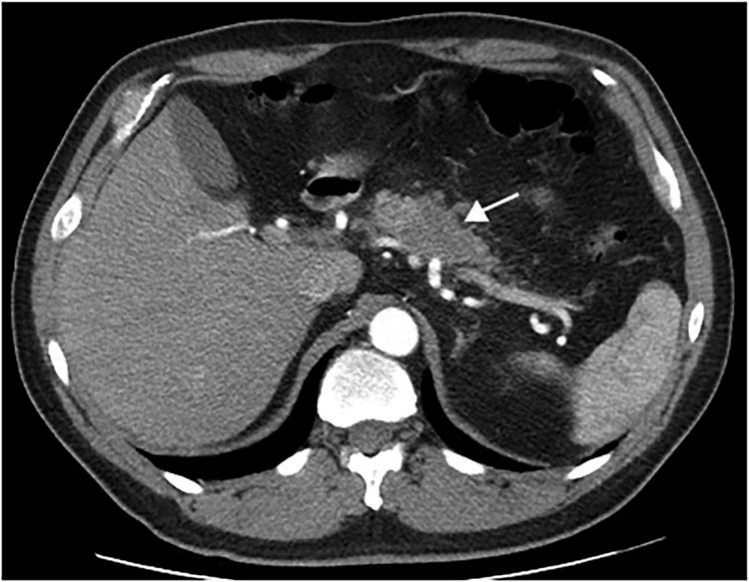
Preoperative CT-scan (arrow in the tumor).

This article presents a case involving a 51-year-old male, smoker, diagnosed with a pancreatic tumor after routine exams revealed an elevated Ca19.9 tumoral marker. The CT scan shown a 4-cm hypodense pancreatic mass with poorly defined borders in the body of the pancreas (Fig. 1). The tumour had interface with vascular structures and no regional or distant metastases were observed. Consequently, surgery was recommended after Multidisciplinary Tumor Board meeting.

In August 2023, the patient underwent elective surgery. It was observed a mass within the pancreatic body, with no vascular invasion or distant metastasis. A laparoscopic distal pancreatectomy with splenectomy was performed and both surgery and postoperative period were uneventful.

**Figure 2:**
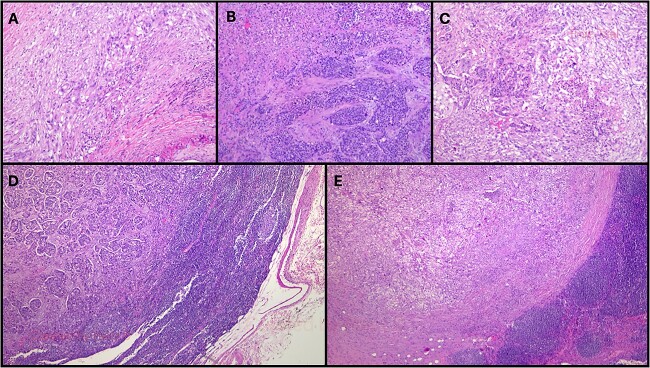
Histopathological examination of this case. (A) More than 60% of the neoplasm was composed of poorly-differentiated ductal adenocarcinoma (HE, original magnification 40×); (B). Over 30% of the neoplasm was composed of a large cell neuroendocrine carcinoma with a preserved organoid pattern (HE, original magnification 40×); (C). Both nonneuroendocrine and neuroendocrine components intermingled in some areas (HE, original magnification 40×); (D). A lymph node metastasis of the nonneuroendocrine component was observed (HE, original magnification 100×); (E). There was also a lymph node metastasis of the neuroendocrine component (HE, original magnification 100×). HE: hematoxylin-eosin.

The histopathological examination (Fig. 2) revealed a 3.7-cm lesion in the pancreas, involving the pancreatic duct, with extension into peripancreatic adipose tissue, compatible with a MiNEN. The non-neuroendocrine component constituted over 50% of the lesion and was identified as a high-grade ductal adenocarcinoma. Meanwhile, the neuroendocrine component accounted for over 30% of the lesion and was characterized as a high-grade large cell neuroendocrine carcinoma with associated necrosis, 22 mitoses/2 mm^2^, a Ki67 index of 50–60%. Lymphovascular and perineurial invasions were noted, and tumoral margins were negative. Fifteen lymph nodes were isolated, with two testing positive: one for neuroendocrine carcinoma metastasis and the other for ductal adenocarcinoma metastasis. The tumor was classified as pT2N1M0.

Following the recommendations of both the Hepatobilio-pancreatic and Endocrine Multidisciplinary Tumor Board, the patient is currently undergoing chemotherapy with FOLFIRINOX.

## Discussion

The authors present the case of a male patient initially diagnosed with a 3.7-cm pancreatic tumor suspected to be a ductal adenocarcinoma. However, subsequent to distal pancreatectomy with splenectomy, the pathology revealed an adeno-MiNEN with lymph node metastasis involving both neuroendocrine and nonneuroendocrine components. This case prompts reflection on the optimal treatment strategy for pancreatic MiNEN and raises the question of how the preoperative diagnosis could influence the patient’s outcome.

Although there was no preoperative suspicion of a MiNEN, the authors believe that the preoperative histological diagnosis was inconsequential in the management of this case, because misdiagnosis doesn’t pose a risk of under-treatment for the MiNEN in this case, given the worse prognosis of the suspected histology, ductal adenocarcinoma [4]. In fact, since this tumor meets anatomic criteria for resectability, radical surgery remains the recommended primary treatment for both neuroendocrine carcinoma and ductal adenocarcinoma [8, 9], consistent, as well, with the treatment provided in published studies on pancreatic adeno-MiNEN [2, 4, 5]. Neoadjuvant treatment is not currently considered for this tumor type due to the lack of available data regarding its impact on prognosis [4], primarily resulting from its rare incidence and challenges in obtaining accurate preoperative histology. Additionally, its effectiveness is questionable in resectable adenocarcinoma and neuroendocrine carcinoma compared to initial surgery [8, 9].

Still, accurate preoperative diagnosis of MiNEN could have value in the metastatic setting, as the chemotherapy regimens should target the metastatic component.

The optimal therapeutic strategies for MiNEN remain uncertain due to the scarcity of high-quality evidence. This is exacerbated by the rarity of this tumor and its tendency to be identified only post-resection. Also, the presence of two distinct histologies with divergent systemic treatment responses adds complexity. The selective treatment of one component may inadvertently favor the clonal expansion of the other, hastening the development of resistance [10]. Given the dismal prognosis associated with high-grade MiNEN, available studies advocate for a multimodal approach for resectable disease, involving surgery followed by adjuvant chemotherapy [4–6, 10]. However, despite the majority of patients received adjuvant chemotherapy [2, 4, 5], its therapeutic benefit remains uncertain [6, 7]. Notably, the only recommendations addressing systemic treatment for MiNEN patients are found in the European Neuroendocrine Tumor Society guidelines [9], suggesting treatment algorithms similar to those applied in pure neuroendocrine carcinoma. Afterwards, DeMeister [6] proposed a standardization of the management of digestive system MiNEN. For high-grade MiNEN, the suggested approach involves surgical resection followed by chemotherapy regimens targeting the component that is assumed to define prognosis, the dominant or most aggressive component. In fact, there is a disparity in the preferential chemotherapy regimens used in the available evidence, reflecting the difficulty of predicting the natural course of disease in adeno-MiNEN given its dependence on not just one but two distinct histologies. In studies by Niessen [2], Frizziero [5, 10] and Oneda [11], most chemotherapy regimens target poorly differentiated adenocarcinoma with gemcitabine or 5-fluorouracil plus irinotecan and/or oxaliplatin. On the contrary, in the studies by Angelico [4], DeMeister [6, 12] and Jacob [13], the neuroendocrine component was the most commonly target.

The authors selected FOLFIRINOX, targeting the non-neuroendocrine component. Despite both components being present in lymph node metastasis, ductal adenocarcinoma carries a worse prognosis than neuroendocrine carcinoma [14]. Furthermore, both ENET and ESMO guidelines for neuroendocrine carcinoma include adenocarcinoma-like regimens with 5-fluorouracil/oxaliplatin/irinotecan [8, 9], since this regimen demonstrated anti-tumor activity for neuroendocrine carcinoma in small retrospective series [10].

In conclusion, this case exposes the complexity of the management of MiNEN and the need for prospective trials to define the best treatment for these patients.
